# The neurobiology of internet addiction: a scoping review of developmental and gender-specific mechanisms

**DOI:** 10.3389/fpsyg.2026.1729470

**Published:** 2026-02-12

**Authors:** Jun Jin, Hanrui Wei, Yan Chai, Linxiao Wu, Mengying Chen, Keya Ding

**Affiliations:** 1 School of Preschool Education, Luoyang Normal University, Luoyang, China; 2 Shanghai Institute of Early Childhood Education, Shanghai Normal University, Shanghai, China

**Keywords:** adolescence, adults, brain structure, gender differences, internet addiction

## Abstract

**Background:**

Internet addiction (IA) constitutes a significant public health challenge, yet the distinct influence of neurodevelopmental stage and gender on its neural substrates remains under-characterized. This scoping review systematically synthesizes neuroimaging evidence to identify convergent brain alterations across IA subtypes and to disentangle the specific neural signatures associated with age and gender.

**Methods:**

Following PRISMA-ScR guidelines, we reviewed peer-reviewed studies published between 2015 and 2025. Searches were conducted across PubMed, Web of Science, and PsycINFO, focusing on investigations that explicitly incorporated age or gender as analytical factors in examining IA-related structural or functional brain changes.

**Results:**

The synthesized evidence indicates that IA is consistently associated with abnormal gray matter volume (GMV), compromised white matter integrity (WMI), and functional dysregulation within the prefrontal cortex (PFC), cingulate gyrus, and reward networks. Developmentally, findings reveal distinct trajectories: adolescents exhibit heightened vulnerability in regions governing emotion regulation and executive control, reflecting a developmental mismatch; in contrast, adults display alterations more indicative of established habit formation and reward circuit remodeling. Gender-specific patterns are also evident: males typically manifest neural alterations linked to gaming behaviors and impulse control, whereas females exhibit distinct profiles involving social–emotional processing networks.

**Conclusion:**

These findings confirm that IA pathology is not uniform but is significantly modulated by demographic factors. The evidence highlights the necessity of shifting from generalized approaches to prevention and intervention strategies that are tailored to specific developmental windows and gender profiles. Future research must prioritize longitudinal and multimodal designs to move from descriptive mapping to causal mechanistic understanding.

## 1 Introduction

The ubiquity of digital technology has precipitated the rise of Internet Addiction (IA), a condition defined by a generalized loss of control and functional impairment (Brand et al., 2019). While constructs including Internet Gaming Disorder (IGD), problematic smartphone use (PSU), and short-video addiction (SVA) are often examined independently, emerging evidence suggests they share overlapping neurobiological substrates (Montag et al., 2021). Consequently, this review adopts IA as an umbrella term to synthesize convergent neural mechanisms.

Despite growing neuroimaging evidence, the literature remains fragmented regarding demographic moderators. Adolescents are uniquely susceptible due to neurodevelopmental immaturity, yet existing studies largely focus on isolated age groups, obscuring the evolutionary trajectory of neural alterations from adolescence to adulthood (Kuss and Griffiths, 2012). Furthermore, while gender differences in prevalence are well-documented, the role gender as a specific moderator of neural vulnerability remains under-characterized (Marraudino et al., 2022). To address these gaps, this scoping review synthesizes empirical research from the past decade, explicitly framing age and gender not merely as covariates, but as theoretical factors that modulate the neural expression of addiction.

### 1.1 Internet addiction as a global health issue

Rapid advances in digital technology have entrenched IA as a global public health concern (Ding et al., 2023). A meta-analysis of 53 studies across 17 countries estimated a global prevalence of 3.05%, with rates significantly higher in Asia (5.1%) than in Europe (2.7%) (Stevens et al., 2021). Crucially, epidemiological data reveal a pronounced developmental vulnerability. In Europe, the prevalence of internet-related disorders among adolescents (12–17 years) is more than double that of young adults (5.8% vs. 2.8%) (Geisel et al., 2021). This disparity is even more acute in Asia, where detection rates among Chinese adolescents reach approximately 19.8% (Li H. et al., 2021). Behavioral data from the United States further substantiate this trend, with nearly 46% of adolescents reporting being online ‘almost constantly’, which reached a twofold increase since 2015 (Chemnad et al., 2025). These findings underscore that IA is not uniformly distributed but disproportionately affects developing populations.

### 1.2 Behavioral and neural effects of internet addiction

Excessive internet use precipitates adverse outcomes across multiple behavioral domains, manifesting as a complex condition rooted in underlying neural dysfunction rather than merely excessive behavior. Physiologically, IA is frequently linked to sedentary lifestyles, reduced physical activity, weight gain, and visual strain, alongside commonly reported comorbidities such as sleep disturbances and circadian rhythm disruptions (Mishra et al., 2024; Xu et al., 2020). Cognitively, accumulating evidence indicates robust associations between IA and impairments in attention, memory, and executive functioning (Ding et al., 2023). Psychiatrically, IA frequently co-occurs with anxiety, depression, and attention-deficit/hyperactivity disorder (ADHD), as well as elevated levels of social anxiety, social avoidance, and obsessive-compulsive personality traits (Marraudino et al., 2022). These extensive functional impairments are underpinned by consistent disruptions across large-scale brain networks, particularly those supporting reward salience, cognitive control, and self-referential processing (Brand et al., 2019; Chang and Lee, 2024).

A central theoretical account posits that hypersensitivity within the reward salience network (SN), involving the ventral striatum and orbitofrontal cortex (OFC), serves as the core mechanism driving pathological craving and reinforcing maladaptive learning processes. This hypothesis is supported by converging multimodal neuroimaging evidence. Structurally, diffusion tensor imaging (DTI) has demonstrated reduced white matter integrity (WMI) in the OFC, which serves as a critical hub for value-based evaluation, among adolescents with IA. Furthermore, structural abnormalities in the thalamus showed a positive correlation with disorder severity (Weinstein, 2017). Complementarily, voxel-based morphometry (VBM) analyses have shown reduced Gray Matter Volume (GMV) in reward-related brain regions among young individuals, reflecting experience-dependent neuroadaptive changes associated with prolonged exposure (Takeuchi et al., 2018). Functionally, functional Magnetic Resonance Imaging (fMRI) studies have demonstrated exaggerated activation of the anterior cingulate cortex (ACC) and OFC in excessive gamers when exposed to gaming-related cues (Han et al., 2010). Notably, these alterations appear to exhibit gender specificity: males with IA have been shown to display significantly greater activation strength and functional connectivity in reward- and emotion-related regions, including the nucleus accumbens (NAcc) and amygdala, compared with females (Hoeft et al., 2008; Wang M. et al., 2019).

In parallel with reward sensitization, deficits in impulse control and behavioral inhibition are closely linked to the dysfunction of the cognitive control network (CNN), centered on the dorsolateral prefrontal cortex (DLPFC) and ACC. Structural evidence integrating fMRI, VBM, and DTI has demonstrated reduced GMV in the DLPFC and ACC among individuals with IA, with these reductions negatively correlated with addiction duration. Concurrently, fractional anisotropy (FA) values in the posterior limb of the internal capsule were found to be positively correlated with addiction duration, suggesting microstructural alterations in white matter tracts induced by chronic usage (Yuan et al., 2011). Electrophysiological and hemodynamic evidence further corroborates these structural deficits. Electroencephalography (EEG) data from Go/NoGo tasks revealed reduced NoGo-N2 amplitudes and increased NoGo-P3 amplitudes with prolonged latency, indicating impaired inhibitory control processes (Dong et al., 2010). More recently, functional near-infrared spectroscopy (fNIRS) studies have confirmed that abnormal prefrontal oxygenated hemoglobin responses during reward processing are significantly associated with symptom severity (Zhang et al., 2025).

Furthermore, the pathology of IA extends to altered interactions between the default mode network (DMN) and the SN, which are increasingly implicated in persistent craving, maladaptive self-referential processing, and emotion regulation difficulties (Chang and Lee, 2024). Structurally, a DTI study indicates elevated FA values in the posterior cingulate cortex (PCC), among some adolescents with IA (Weinstein, 2017), while VBM analyses have revealed significant reductions in cerebellar GMV, a region recognized for its role in emotion regulation and its interaction with DMN-related processes (Yuan et al., 2011). Functionally, fMRI studies demonstrate significantly reduced connectivity among the DMN, Executive Control Network (ECN), and SN. This disrupted network coordination undermines the integrated functioning of self-referential processing, cognitive control, and motivational salience, thereby exacerbating craving and emotional dysregulation (Chang and Lee, 2024).

### 1.3 Age-and gender-related vulnerability to internet addiction

Neurobiological susceptibility to IA varies substantially across the lifespan, reflecting non-linear developmental trajectory of brain. The Dual Systems Model posits that in adolescence, the early maturation of subcortical reward regions outpaces the development of prefrontal inhibitory control, resulting a “developmental mismatch” that heightens addiction risk (Mills et al., 2016). In contrast, IA in adult may reflect different mechanisms, such as habit formation or chronic stress adaptations, given their relatively mature executive systems (Koob and Volkow, 2016).

Gender acts as another critical moderator. Behavioral data suggest males are prone to gaming-related addiction, while females show higher susceptibility to social media dependency and associated emotional distress (Kuss and Griffiths, 2017). These behavioral phenotypes map onto distinct neural signatures: males exhibit prominent reward-circuit alterations, whereas females show stronger involvement of emotion-processing and self-referential networks (Sadeghi et al., 2021). However, integrative research systematically comparing these groups remains scarce.

### 1.4 Research gaps and the necessity of this review

Despite the proliferation of neuroimaging studies, the current literature is characterized by significant heterogeneity in age composition, gender representation, and diagnostic criteria (Horvath et al., 2020). Most existing reviews treat age and gender as noise to be controlled rather than as signal to be analyzed. Given this complexity, a scoping review is the optimal approach to map the breadth of evidence and identify transdiagnostic patterns.

This review systematically synthesizes structural and functional neuroimaging findings from the past decade to answer four key questions:

1. Which functional and structural alterations are consistently associated with IA across different subtypes?
2. How do these neural alterations differ between adolescents and adults?
3. What specific patterns of functional connectivity characterize IA across developmental stages?
4. How does gender moderate the relationship between IA subtypes, neural correlates, and behavioral risks?

## 2 Materials and methods

The rapid advancement of neuroimaging techniques has catalyzed a proliferation of empirical research examining the neural substrates of IA. To provide a comprehensive synthesis of this expanding field, the present review integrates evidence from the past decade. The methodological approach was grounded in the five-stage framework outlined by Arksey and O'malley (2005) and adhered to the Preferred Reporting Items for Systematic Reviews and Meta-Analyses—Scoping Review (PRISMA-ScR) guidelines. Specifically, we systematically engaged in identifying, selecting, charting, and synthesizing data to delineate the structural and functional brain alterations associated with IA across adolescent and adult populations. This systematic mapping aims to provide a robust evidence base for elucidating underlying mechanisms and informing early identification and intervention strategies.

### 2.1 Identifying relevant studies

A systematic literature search was conducted across three major electronic databases: PubMed, Web of Science, and PsycINFO, concluding on May 10, 2025. The search strategy queried title, abstract, and keyword fields using a combination of terms covering both broad constructs of digital addiction (e.g., Internet addiction) and specific high-prevalence subtypes (e.g., short-video). This broad scope ensured the retrieval of studies relevant to the central theme of Internet/digital addiction and its neural correlates. Regarding diagnostic criteria, given the ongoing debate and lack of consensus in the field, we did not impose a standardized diagnostic threshold *a priori*. Instead, we adopted an inclusive approach, extracting and documenting the specific definitions and psychometric tools exactly as applied in the primary studies. This allowed for a nuanced interpretation of findings within their respective conceptual frameworks, ensuring that methodological heterogeneity was transparently acknowledged.

Relevant studies were identified using a Boolean search strategy combining keywords related to digital media subtypes, addiction constructs, impact assessment, and neuroimaging modalities. The specific search string was constructed as follows: (“Short-Video” OR “TikTok” OR “Phone” OR “Internet” OR “Web” OR “WeChat”) AND (“addiction” OR “indulge” OR “immerse” OR “excessive”) AND (“influence” OR “effect” OR “affect” OR “impact”) AND (“Brain structure” OR “Brain function” OR “Prefrontal Cortex” OR “EEG” OR “fNIRS” OR “fMRI” OR “MRI”) AND (“adults” OR “college student” OR “undergraduate” OR “adolescence” OR “children”).

To ensure the retrieval of high-quality, contemporary evidence, filters were applied for publication date (January 1, 2015, to May 10, 2025) and English language. Notably, restrictions on document type or participant age were intentionally omitted during the initial search phase to maximize sensitivity and prevent the premature exclusion of relevant literature.

The search yielded a total of 3,027 records distributed across PubMed (*n* = 1,102), Web of Science (*n* = 1,453), and PsycINFO (*n* = 472). These databases were selected for their exhaustive coverage of biomedical, psychological, and interdisciplinary neuroimaging literature. Web of Science was prioritized over Scopus due to its rigorous indexing standards for core journals in the life and social sciences. This strategic selection aimed to capture high-impact research while minimizing redundancy across databases. All subsequent screening was performed manually according to the rigorous inclusion and exclusion criteria outlined below.

### 2.2 Studies selection

During the full-text screening phase, the Science Citation Index indexing status of journals was verified by consulting official websites or authoritative databases (e.g., the Master Journal List by Clarivate Analytics). Restricting inclusion to SCI-indexed journals ensures rigorous peer review and standardized reporting, which is crucial for maintaining the methodological validity of neuroimaging studies involving complex data analysis.

During synthesis, the operational definitions of age and gender were based on explicitly reported classifications within the included studies. For age, findings were categorized based on mean age or ranges defined by the original authors (typically defined as adolescents: 10–18 years; adults: 18–30 years) to encompass critical neurodevelopmental stages from early adolescence to early adulthood. This facilitated the examination of brain changes during key periods related to IA susceptibility, such as PFC maturation and reward system development. For gender, classifications were based on biological sex or reported social identity (categorized as male or female).

To ensure the rigor of the review, specific inclusion and exclusion criteria were established. Studies were included if they met the following criteria: (1) were peer-reviewed research papers; (2) were published between 2015 and May 2025; (3) were published in journals indexed in the SCI; (4) were written in English; (5) included participants aged between 10 and 30 years; (6) utilized neuroimaging techniques (e.g., fMRI, fNIRS, EEG) or reported specific brain region measurements; (7) identified the experimental group with at least one type of addictive behavior (e.g., IA, Smartphone Addiction [SPA], Social Networking Site Addiction [SNSA], SVA); and (8) focused on the effects of addictive behaviors on brain function or structure.

Studies were excluded if they were: (1) reviews, theoretical studies, editorials, or secondary data analyses; (2) non-peer-reviewed articles; (3) published in languages other than English; (4) focused on participants outside the defined age range (<10 or >30 years); or (5) lacked specific diagnostic criteria for addiction or neuroimaging data.

**Figure 1:**
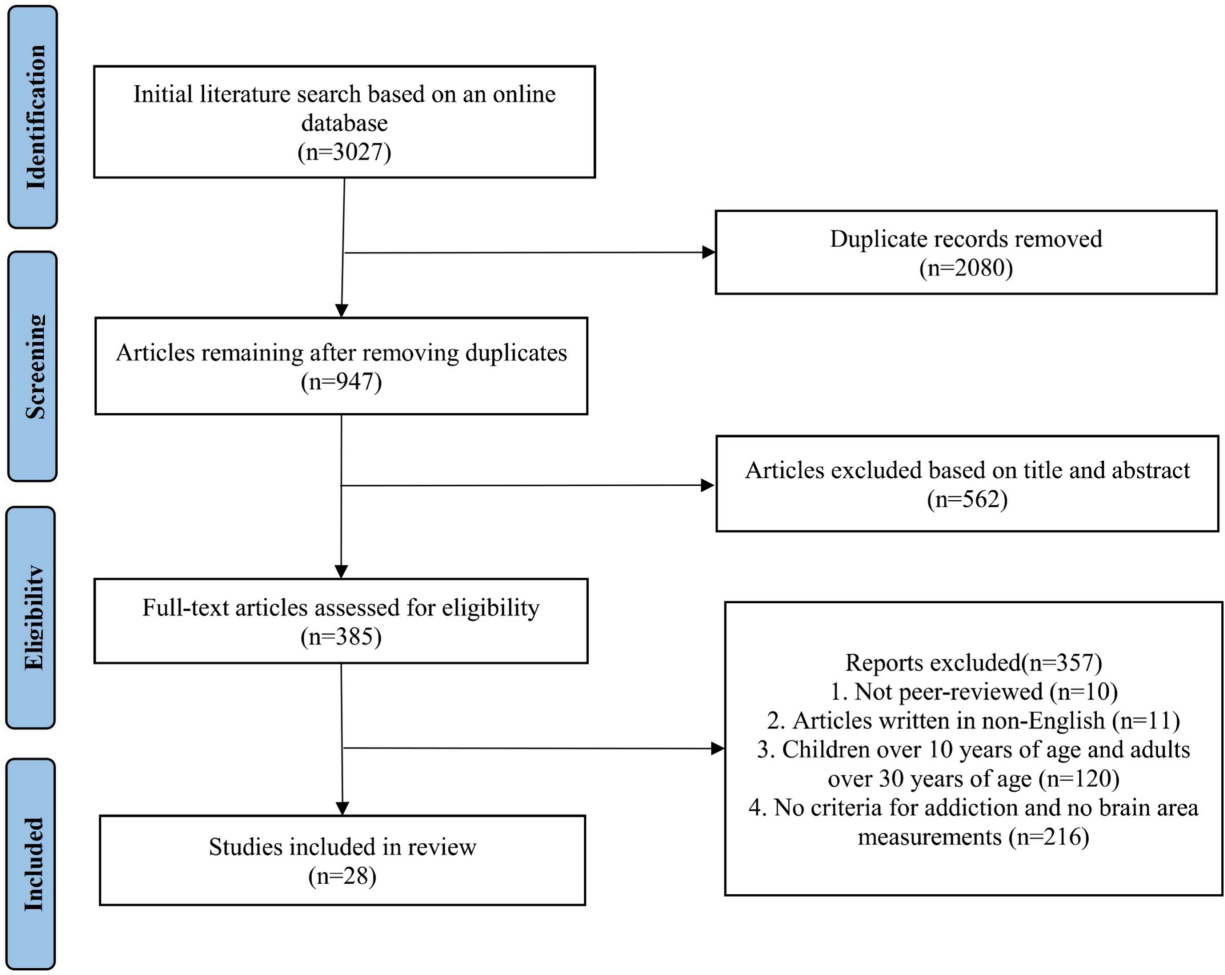
PRISMA flow diagram of the study selection process. This diagram details the sequential stages of the literature screening process, from the initial identification of records in electronic databases to the final inclusion of eligible studies. It delineates the specific flow of information through the phases of identification, deduplication, title/abstract screening, and full-text eligibility assessment, along with the reasons for exclusion at each step.

The screening process proceeded as follows: First, EndNote was used to remove 2,080 duplicate references, leaving a total of 947 articles. Subsequently, 562 articles were excluded based on title and abstract screening. Full-text review was then conducted, resulting in the exclusion of articles based on the following reasons: (1) not peer-reviewed (*n* = 10); (2) not written in English (*n* = 11); (3) participants outside the age range of 10–30 years (*n* = 120); and (4) lack of addiction criteria or brain measurements (*n* = 216). Ultimately, 28 eligible articles were included in the scoping review. To ensure validity, three reviewers cross-coded the data and reached a consensus on the final selection. This study adheres to the PRISMA-ScR guidelines (Figure 1).

### 2.3 Charting date

To ensure rigor, the research team developed and piloted standardized data extraction forms prior to full implementation. Data extraction was performed independently by two trained reviewers, with any discrepancies resolved through consensus discussions involving a third reviewer. The specific inclusion and exclusion criteria established for this review are detailed in Table 1.

**Table 1:** Inclusion and exclusion criteria.

Criterion	Inclusion	Exclusion
Scope of research	Empirical studies	Not empirical studies (reviews, theoretical, studies, editorials); Secondary data analysis
Type of documents	Peer-reviewed research articles	Non-peer-reviewed articles
Period	March 2015–May 2025	Before 2015 and after July 2025
Language	English	Languages other than English
Participant	Children and Adults aged are between 10 and 30 years old	Children are under 10 years old, and adults are over 30 years old
Addition Type	Identified with at least one type of addictive behavior (SVA, IA, SNSA, SPA)	No mention of anything related to addiction and none of the addictive behaviors are judged
Method	Brain imaging techniques (fMRI, fNIRS, EEG) or involving brain regions	Only behavioral tests, no brain areas involved
Topic	The effects of certain addictive behaviors on brain function or brain structure	Article does not cover brain functionor brain structure

SVA, short-video addiction; IA, internet addiction; SNSA, social network site addiction; SPA, smartphone addiction; fMRI, functional magnetic resonance imaging; fNIRS, functional near-infrared spectroscopy; EEG, electroencephalography.

Furthermore, a comprehensive summary of the 28 eligible articles is presented in the Supplementary material. This summary categorizes extracted data into three key domains: (1) bibliographic and demographic information (e.g., author, year, country, sample size, age range); (2) methodological parameters (e.g., addiction subtypes/criteria, neuroimaging modalities, behavioral paradigms); and (3) key research findings, including specific brain alterations and reported limitations.

### 2.4 Collating, summarizing, and reporting

A narrative synthesis approach was adopted to interpret the findings. Initially, the 28 included studies were stratified by neuroimaging modality (i.e., fNIRS, Magnetic Resonance Imaging [MRI], fMRI, EEG, and DTI). To facilitate meaningful integration across these diverse techniques, our analysis prioritized converging evidence regarding brain systems (e.g., the prefrontal executive network, reward circuitry) and associated behavioral domains (e.g., cognitive control, decision-making), rather than relying on direct comparisons of modality-specific metrics. Accordingly, the synthesis was organized around three key themes: (1) demographic characteristics of the studied populations; (2) structural and functional brain alterations associated with IA; and (3) developmental distinctions in neural patterns between adolescents and adults. Detailed study characteristics are provided in Table 2 (and the Supplementary material).

**Table 2:** Distribution of studies by country and year of publication.

Country	Number	Year of publication
China	17	2015, 2017(3), 2018, 2019, 2021(2), 2023, 2024(3), 2025(5)
Germany	2	2020, 2022
Italy	2	2017
Republic of Korea	2	2018, 2023
United States	1	2020
Austria	1	2024
Hungary	1	2019
Iran	1	2021
Japan	1	2018

Regarding methodological limitations, we acknowledge the potential for publication bias (favoring positive results) and language bias (restriction to English-language literature). Consistent with the methodological framework of Arksey and O'malley (2005), which prioritizes mapping the breadth and nature of available evidence over assessing methodological quality, a formal risk-of-bias assessment was not conducted.

## 3 Results

### 3.1 Study characteristics and scope of evidence

#### 3.1.1 Demographic information

The 28 articles included in this review were published between March 2015 and May 2025. More than half appeared in the past 5 years, reflecting increasing research interest in this field. Geographically, most studies originated from Asia and Europe. China contributed the highest number of articles (*n* = 17), followed by Germany, Italy, and South Korea (2 studies each). The dominance of Asia, particularly China, may stem from the region’s heightened public health concern regarding IA and substantial research investment in this field.

A cumulative total of 6,687 participants were aggregated across the 28 included studies. Regarding developmental stratification, 8 studies specifically investigated adolescent populations (aged 10–18 years), while the remaining 20 focused on young adults (aged 18–30 years). Methodologically, the literature relies heavily on cross-sectional designs; 27 studies were exclusively cross-sectional, with only a single study incorporating a longitudinal component. Detailed participant characteristics (e.g., sample size, group distribution, and gender ratios) are provided in Supplementary material.

#### 3.1.2 Research paradigm and addiction subtypes

Of the 28 included studies, 13 utilized task-based paradigms to probe specific cognitive domains. The most frequently targeted processes were reward processing and risk decision-making (*n* = 3; i.e., Balloon Analogue Risk Task, Mixed Gambling Task, Open-box Continuous Risk Task) and inhibitory control (*n* = 3; i.e., Go/No-Go, Stop-Signal Task). Additional domains included working memory (N-back), attentional control (Attention Network Test, Stroop task), creativity (Alternate Uses Task), and passive video viewing. Notably, one study employed a composite approach, combining a modified Operation Span Task and Task Switching Paradigm to assess broader executive functions.

Regarding the classification of addiction subtypes across the full sample (*N* = 28), investigations predominantly focused on generalized IA and SPA (*n* = 9 each; 32.1%), followed closely by SVA (*n* = 8; 28.6%). The SNSA represented a smaller proportion of the literature (*n* = 2; 7.1%) (Table 3). This distribution reflects a multidimensional approach to investigating IA. It also highlights the current emphasis on high-prevalence digital behaviors, particularly internet and smartphone use, and the growing attention to platform-specific behaviors such as SVA.

**Table 3:** Types of addiction in the 28 studies.

Types of addiction	Number
Internet Addiction	9
Smartphone Addiction	9
Social Network site Addiction	2
Short Video Addiction	8

#### 3.1.3 Neuroimaging technique

The neuroimaging techniques used in the 28 studies are shown in Table 4. Seven studies employed EEG, 7 used fMRI, 3 utilized fNIRS, and 1 used DTI. The remaining 10 studies applied multimodal MRI approaches. Specifically, these combined methods included structural MRI with fMRI (*n* = 5), MRI with VBM (*n* = 3), MRI with DTI (*n* = 1), and a comprehensive combination of fMRI, DTI, VBM, and MRI (*n* = 1).

**Table 4:** Types of neuroimaging technique in the 28 studies.

Types of neuroimaging technique	Number
fNIRS	3
fMRI	7
DTI	1
EEG	7
MRI and VBM	3
fMRI and MRI	5
MRI and DTI	1
fMRI DTI VBM and MRI combined	1

fNIRS, functional near-infrared spectroscopy; fMRI, functional magnetic resonance imaging; DTI, diffusion tensor imaging; EEG, electroencephalography; MRI, magnetic resonance imaging; VBM, voxel-based morphometry.

#### 3.1.4 The brain regions of internet and digital addiction

This review synthesized 28 neuroimaging studies published between 2015 and 2025 to examine the neural correlates of internet and digital addiction using EEG, fNIRS, fMRI, DTI, MRI and multimodal methods.

The included studies revealed functional and structural brain abnormalities concentrated primarily in large-scale neural networks and specific brain regions. These regions broadly fall into two clusters. The first is located in the anterior brain, primarily involving the prefrontal-limbic circuitry; the second is situated in the posterior brain, involving core regions of the DMN. The anterior cluster includes the prefrontal cortex (DLPFC, dorsomedial prefrontal cortex [dmPFC], ventromedial prefrontal cortex, and OFC); the ACC (dorsal anterior cingulate cortex, subgenual anterior cingulate cortex); and the limbic and parahippocampal systems (amygdala, NAcc, hippocampus, and parahippocampal gyrus [PHG]). The posterior cluster primarily includes the PCC/precuneus and the subcuneate lobule/angular gyrus.

### 3.2 Functional brain alterations

#### 3.2.1 Altered activation in core neurocognitive systems

##### 3.2.1.1 Default mode network

Across the spectrum of IA subtypes, including SVA, SPA, and SNSA, neuroimaging evidence converges on a consistent profile of aberrant DMN function. This dysfunction is primarily characterized by hyperactivation during resting states and deficits in task-induced deactivation. Anatomically, these alterations are anchored in key DMN hubs, including the medial prefrontal cortex (mPFC), PCC, dmPFC, and extending to the OFC. Theoretically, this pattern reflects an excessive engagement in introspective thinking and maladaptive self-referential processing, a neural signature that parallels the behavioral phenotype of compulsive mental preoccupation.

Resting-state analyses reveal a landscape of DMN hyper-connectivity. Wang Y. et al. (2019) demonstrated increased local functional connectivity density within the PCC, which correlated positively with addiction severity. This hyper-connectivity is corroborated by findings of increased activity in DMN nodes (e.g., DLPFC, PCC) and the temporoparietal junction (TPJ) in SVA populations (Gao et al., 2025). Furthermore, Schmitgen et al. (2022) highlighted an altered inter-network balance in SPA, suggesting a complex reorganization of resting-state dynamics where parietal non-DMN regions may show compensatory enhancement while specific prefrontal nodes exhibit reduced fluctuations.

Crucially, IA pathology extends beyond rest to a failure of task-induced deactivation. Normatively, the DMN should suppress activity during cognitively demanding exogenous tasks to facilitate focused attention. However, individuals with IA exhibit an attenuated suppression of the DMN during focused attention (Darnai et al., 2019) and working memory tasks (Saeid Sadeghi et al., 2021). This persistent DMN “intrusion” likely interferes with executive resources, reflecting a deficit in the antagonistic toggling between internal and external attention systems. This failure of suppression is pervasive, extending even to contexts involving risk decision-making and reward processing (Liu et al., 2023; Zhang and Li, 2025), indicative of widespread network-level inefficiency.

DMN dysfunction also underpins the heightened reactivity to addiction-related cues. Exposure to personalized triggers (e.g., algorithmic video recommendations) elicits robust activation in the mPFC, PCC, and temporal poles (Li Z.-L. et al., 2021; Su et al., 2021). This suggests that addiction cues are not merely processed as external stimuli but are integrated into self-referential schemas. This bias is further evidenced by altered fronto-parietal activation (Balconi et al., 2017a) and weakened connectivity between the ECN and DMN during creative cognition (Li et al., 2023), highlighting the decoupling of cognitive control from self-regulation.

In the context of neurodevelopment, DMN alterations in adolescents appear particularly linked to emotional and social processing. Enhanced functional connectivity in DMN-related regions (e.g., insula, middle temporal gyrus) has been observed in adolescents with IA, involving areas critical for emotional integration (Ko et al., 2023). Uniquely, these alterations often mediate pathways involving negative self-perception, such as bullying victimization (Yao et al., 2025), and are associated with broader emotional dysregulation (Lin et al., 2015). This suggests that for adolescents, DMN dysfunction may represent a specific vulnerability in the integration of social identity and emotional regulation.

##### 3.2.1.2 Cognitive control network

Evidence form seven neuroimaging studies consistently points to a functional compromise within the CCN, particularly involving the DLPFC and ACC. Generally, this dysfunction manifests as hypoactivation and functional decoupling during standard cognitive demands. Specifically, attenuated DLPFC activation has been observed during inhibitory control tasks (Li et al., 2021) and creative cognition (Li et al., 2023), correlating with behavioral deficits in attention and executive flexibility. This localized blunting is further supported by Schmitgen et al. (2022), who reported global reductions in low-frequency fluctuations across the medial PFC and DLPFC during both resting and task states. Beyond focal activation, network integrity appears compromised: Darnai et al. (2019) identified a generalized failure to recruit the inhibitory control network during the Stroop task, while connectivity analyses reveal a decoupling of the ACC from the insula and parietal lobules in SPA populations (Juliane Horvath et al., 2020; Tie et al., 2025). Collectively, these findings suggest a systemic failure in top-down regulation.

Electrophysiological evidence from four EEG studies further elucidates the temporal dynamics of this impairment. In adults, aberrant oscillatory patterns underscore deficits in information processing and synchronization. Yan et al. (2024) linked reduced prefrontal theta power to weakened attentional monitoring, while Balconi et al. (2017a) observed excessive delta/theta activity in parietal–frontal circuits during inhibition failures. Similarly, Xu et al. (2024) found that severity of SVA correlated with blunted temporal-occipital delta enhancement, indicative of disrupted working memory synchronization. Developmentally, adolescents exhibit unique markers of immaturity: Tseng et al. (2024) revealed that adolescents with SVA show enhanced prefrontal theta synchrony but reduced beta desynchronization during inhibition. This pattern suggests that the developing cognitive control system is particularly susceptible to ‘locking’ into inefficient oscillatory states, hindering rapid cognitive switching.

Conversely, a distinct pattern of prefrontal hyperactivation emerges specifically during high-stakes or loss-related decision-making. Unlike the hypoactivation seen in standard control tasks, four studies report abnormally heightened PFC engagement when individuals with IA face potential losses. Zhang and Li (2025) and Liu et al. (2025) observed excessive activation in the OFC and anterior cingulate-parietal networks during loss processing in risk tasks. Similarly, heightened recruitment of the DLPFC and OFC has been documented during high-risk scenarios (Liu et al., 2023; Wang M. et al., 2019). Rather than indicating superior control, this paradoxical hyperactivity likely reflects inefficient neural processing, requiring compensatory cognitive effort to regulate impulse, or a hypersensitivity to negative outcomes (loss aversion) that triggers an exaggerated executive response.

##### 3.2.1.3 Reward and emotion processing systems

Evidence from seven studies substantiates a state of mesolimbic hypersensitivity in individuals with IA, characterized by aberrant neural and physiological responses to reward cues. Specifically, Su et al. (2021) demonstrated that algorithmic personalization amplifies this response, reporting significantly heightened activation in the Ventral Tegmental Area (VTA) when participants viewed recommended short videos compared to standard content. This neural sensitization is corroborated by physiological evidence: Balconi et al. (2017a) observed enhanced skin conductance responses during gambling tasks, indicative of heightened autonomic arousal and a reward bias. At the network level, Chun et al. (2018) identified increased functional connectivity between the Middle Cingulate Cortex (MCC) and the NAcc, suggesting a strengthening of the pathway linking motivation to reward seeking.

In adolescents, reward dysfunction is distinctively intertwined with neurodevelopmental vulnerabilities in emotional regulation. Unlike the consolidated circuitry seen in adults, the adolescent brain exhibits a regulatory mismatch. Ko et al. (2023) revealed enhanced negative connectivity between the left NAcc and right cerebellum, while Lin et al. (2015) demonstrated widespread reduced functional connectivity between the ventral striatum and key emotional-motivational nodes (e.g., amygdala, caudate head, and subgenual ACC). Structurally, excessive internet use appears to disrupt normative maturation. Takeuchi et al. (2018), using VBM, found that high-frequency internet use correlates with reduced GMV in the insula, amygdala, and OFC. This suggests that IA may induce maladaptive structural remodeling in regions critical for emotion integration. Conversely, Tymofiyeva et al. (2020) observed enhanced structural connectivity in the right amygdala, potentially reflecting a hypertrophy of threat/stress processing circuits.

Furthermore, five studies elucidate the reciprocal relationship between IA, negative affect, and social stress, supporting a negative reinforcement hypothesis. Behaviorally, IA severity is strongly correlated with anxiety, depression, and loneliness (Li et al., 2021), and withdrawal symptoms are associated with elevated cortisol levels (Chun et al., 2018), marking a physiological stress response. Neurally, this interaction mediates the pathway from social stress to addiction. Tie et al. (2025) and Yao et al. (2025) demonstrated that stress weakens self-control mechanisms, while negative emotions mediate the impact of bullying victimization on SVA severity. Crucially, gender-specific mechanisms are evident, Sadeghi et al. (2021) identified increased GMV in the left anterior insula specifically among females. Given the role of insula in interoception and emotional awareness, this structural alteration provides a biological basis for the heightened link between emotional dysregulation and IA observed in female populations.

##### 3.2.1.4 Feedback and attention-related potentials

Event-Related Potential (ERP) evidence from five studies elucidates the temporal dynamics of impaired outcome evaluation and attentional resource allocation in IA. Regarding feedback processing, adults with IA exhibit a dissociation between early and late evaluation stages. Balconi et al. (2017b) reported significantly attenuated Feedback-Related Negativity amplitudes, suggesting a deficit in the early, semi-automatic detection of negative outcomes (prediction error). Conversely, the subsequent P300 response to feedback was enhanced, indicating a heightened, potentially compensatory, late-stage emotional allocation to reward outcomes.

In the domain of attentional control, abnormalities in the P300 component, an index of context updating and resource allocation, are prevalent. Walla and Zheng (2024) observed blunted P300 amplitudes in the left parietal region during a visual Oddball paradigm in SVA participants, reflecting deficits in selective attention. Similarly, Balconi et al. (2017a) demonstrated that individuals with IA exhibit prolonged P300 latencies and aberrant amplitudes during Go/NoGo tasks. These ERP anomalies were correlated with disrupted theta/alpha phase synchronization in fronto-parietal networks, pointing to a failure in the temporal integration required for inhibitory control.

Developmentally, adolescent ERP profiles suggest a reliance on inefficient, high-effort neural strategies. Xu et al. (2024) linked P300 abnormalities in adolescents to impaired sensory integration. Most notably, Tseng et al. (2024) found that during stop-signal tasks, adolescents with SVA exhibited heightened Stop-P3 amplitudes that exceeded adult levels. This over-activation suggests that the immature adolescent brain requires compensatory neural recruitment to execute inhibitory control, reflecting a state of high developmental plasticity but low processing efficiency compared to the mature adult system.

### 3.3 Functional connectivity alterations

Synthesis of 14 studies reveals that IA is not merely a focal disorder but a systemic pathology involving widespread abnormal functional connectivity. The overarching profile is characterized by disrupted coordination among the ‘Triple Networks’: the DMN, the CCN, and the SN. Evidence suggests a widespread alteration in the interaction between these core systems. Specifically, Liu et al. (2025) and Wang Y. et al. (2019) observed abnormal functional connectivity linking the CCN (e.g., IFG, ACC) with the DMN and reward circuits (striatum, insula). This manifests as an inability of the SN to appropriately regulate the balance between internal processing and external control. For instance, Darnai et al. (2019) described a network imbalance characterized by simultaneous DMN hyperactivity and SN hyper-responsiveness. Complementing this, Sadeghi et al. (2021) noted aberrant DMN engagement even during working memory tasks, indicating a failure to modulate network activity according to task demands.

Abnormal connectivity extends to specific regulatory axes beyond the core triple networks. Regarding the reward-control axis, Ko et al. (2023) identified altered connectivity between the OFC, NAcc, and MCC, which correlated directly with withdrawal severity. In terms of global integration hubs, Tie et al. (2025) revealed reduced connectivity in SPA between the frontoparietal network and multiple sensory/motor modules, as well as between the ACC and the angular gyrus. Similarly, Li et al. (2023) reported disrupted connectivity within the prefrontal-temporal network (DLPFC-TPJ), reflecting a decline in cross-regional information integration capacity.

Distinct neurodevelopmental trajectories underscore the connectivity profiles of adolescents compared to adults. In adolescents, abnormalities are widespread and structurally anchored, predominantly affecting the cortico-striatal circuitry. Lin et al. (2015) identified comprehensive reduced connectivity between the striatum and cognitive control systems, while Chun et al. (2018) conversely reported enhanced connectivity between the OFC/MCC and NAcc. These opposing patterns indicate a fundamental imbalance in the motivation-control pathways. Furthermore, Tymofiyeva et al. (2020) found abnormal nodal centrality in the right amygdala, suggesting that emotion-processing hubs are disproportionately influential in the adolescent network topology. Spontaneous abnormalities are also evident, with Yao et al. (2025) and Tseng et al. (2024) observing abnormal spontaneous activity in medial temporal regions and aberrant synchronization in occipito-parietal networks, indicating that the immature brain is prone to widespread instability. In contrast, adults exhibit a profile characterized by reduced processing efficiency in established networks. Adult alterations typically manifest as abnormal connectivity in specific functional loops required for habit regulation and higher-order control (Darnai et al., 2019; Liu et al., 2025), reflecting deficits in network efficiency rather than the widespread developmental reorganization seen in adolescents.

### 3.4 Structural brain alterations

#### 3.4.1 Gray matter volume alterations

Structural MRI evidence from 10 studies delineates a pattern of GMV alterations associated with IA, characterized by system-specific localization and distinct developmental trajectories.

In regions subserving reward processing and executive control, volumetric changes are prominent. Gao et al. (2025) identified increased GMV in the OFC and bilateral cerebellum in individuals with SVA, suggesting structural adaptations related to reward valuation. Similarly, He et al. (2017) demonstrated a positive correlation between SNSA severity and GMV in the ACC and MCC. Conversely, Montag et al. (2018) reported a dissociation in WeChat addiction, characterized by reduced NAcc volume paired with increased OFC volume, pointing to a structural imbalance in the fronto-striatal reward loop.

The emotional regulation network also exhibits significant volumetric anomalies. Tymofiyeva et al. (2020) and He et al. (2017) consistently linked IA to volumetric alterations in the bilateral amygdala. Notably, gender-specific structural adaptations were observed by Sadeghi et al. (2021), who found increased GMV in the left anterior insula specifically in females. Given the insula’s role in interoception, this suggests a sex-specific biological basis for the emotional dysregulation often seen in IA.

A profound developmental divergence exists between age groups. In adolescents, structural changes appear to reflect a widespread developmental impediment. Takeuchi et al. (2018) revealed that excessive internet use is associated with delayed maturation across broad cortical and subcortical systems, encompassing language processing (superior temporal gyrus), executive function (PFC, ACC, basal ganglia), emotion (insula, amygdala), and memory circuits (hippocampus). This implies that IA may arrest the normative “pruning” or growth processes during critical adolescent windows. In contrast, adults exhibit more focal, experience-dependent plasticity. For instance, Sadeghi et al. (2021) identified increased GMV in the right superior (PHG during working memory tasks), while Horvath et al. (2020) observed a mixed profile in SPA, with reduced GMV in the anterior insula and inferior temporal lobe but increased volume in the superior PHG. These adult patterns likely reflect localized remodeling rather than global developmental delay.

#### 3.4.2 White matter integrity and structural connectivity

Beyond gray matter, alterations in white matter microstructural integrity suggest compromised neural transmission efficiency in IA. Hu et al. (2017) identified impaired WMI in SPA, manifested as reduced FA and increased Mean Diffusivity (MD) in key projection pathways, including the right superior longitudinal fasciculus, the anterior limb of the internal capsule, and the corona radiata. Crucially, addiction severity was negatively correlated with FA and positively correlated with MD, indicating a dose–response relationship between IA severity and fiber tract disruption.

In terms of structural connectivity networks, developmental nuances again emerge. Among adolescents, Tymofiyeva et al. (2020) found that SVA was associated with enhanced structural connectivity to the right amygdala, potentially reinforcing the emotional salience of addictive cues. Regarding specific subtypes like SNSA, evidence also points to macrostructural deficits, with observed reductions in White Matter Volume (WMV) in the Inferior Frontal Gyrus (IFG), lower cerebellum, and temporal lobe regions, further implicating the structural decline of inhibitory and sensory integration networks.

### 3.5 The prefrontal cortex as a convergent neural target

**Figure 2:**
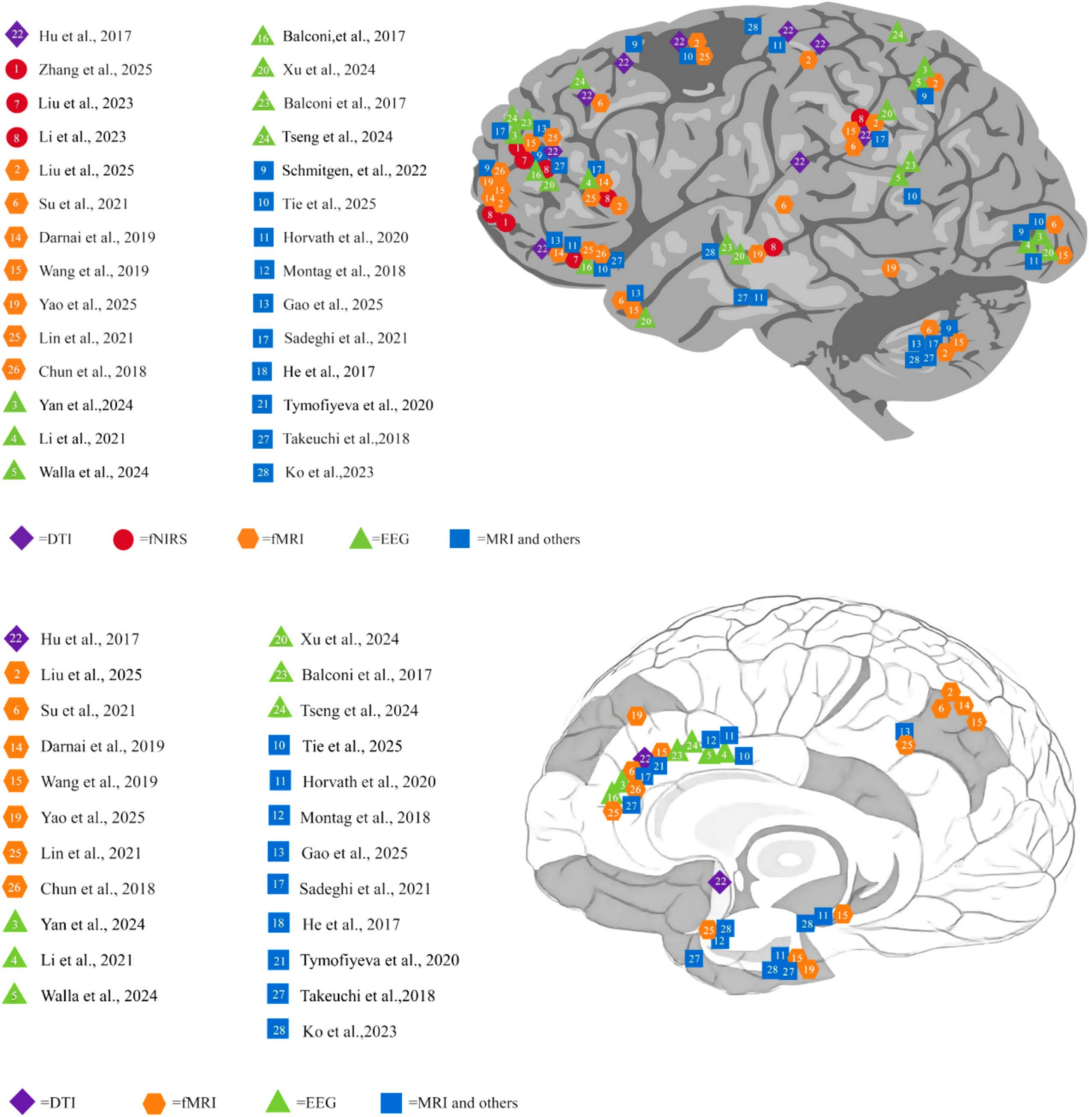
Neuroimaging findings on the effects of Internet addiction on the brain. This figure synthesizes neuroimaging findings from 28 studies, mapping the distribution of brain structural and functional alterations associated with IA across different neuroimaging modalities (DTI, fNIRS, fMRI, EEG, MRI, and combined techniques). Each marker corresponds to a specific study, with colors distinguishing imaging methods to illustrate the consistency and specificity of brain region involvement (e.g., prefrontal cortex, reward-related regions, default mode network) across addiction types (Internet, smartphone, short video, social network addiction) and age/gender groups (Zhang and Li, 2025; Liu et al., 2025; Yan et al., 2024; Li et al., 2021; Walla and Zheng, 2024; Su et al., 2021; Liu et al., 2023; Li et al., 2023; Schmitgen et al., 2022; Tie et al., 2025; Horvath et al., 2020; Montag et al., 2018; Gao et al., 2025; Darnai et al., 2019; Wang M. et al., 2019; Balconi et al., 2017a,b; Sadeghi et al., 2021; He et al., 2017; Yao et al., 2025; Xu et al., 2024; Tymofiyeva et al., 2020; Hu et al., 2017; Tseng et al., 2024; Lin et al., 2015; Chun et al., 2018; Takeuchi et al., 2018; Ko et al., 2023).

A synthesis of evidence from the 28 included studies (2015–2025) identifies the Prefrontal Cortex (PFC) as the undisputed epicenter of neural maladaptation in IA. Across diverse addiction subtypes (SVA, SPA, SNSA) and developmental stages, systematic abnormalities converge on key PFC subregions—notably the DLPFC, OFC, and ACC (Table 5; Figure 2). These alterations constitute the neural substrate for the core behavioral phenotype of the disorder: deficits in cognitive control, aberrant decision-making, and impaired impulse regulation.

**Table 5:** Detailed information on 28 studies.

Modality	Studies	Neural/brain region	Key finding	Behavioral impact	Age effect	Sex effect
EEG	Li et al. (2021) and Walla and Zheng (2024)	Parietal, SN, Temporal, Occipital	Hyperactivation, abnormal P300	Impaired attention	—	Females: ↑behavioral susceptibility
	Yan et al. (2024), Balconi et al. (2017a), Xu et al. (2024), Balconi et al. (2017b), and Tseng et al. (2024)	Prefrontal, Parietal, Temporal, Occipital	↑↓Theta/Delta/Alpha, abnormal P300	↓Cognitive control, ↓WM	Adolescents: ↑sensitivity, Stop-P3 ↑	—
fMRI	Liu et al. (2025), Su et al. (2021), Darnai et al. (2019), and Wang M. et al. (2019)	Cerebellum, DMN, TP, FPA	Hyperactivation, abnormal connectivity	Impaired decision-making, ↓cognitive control	—	—
	Yao et al. (2025), Lin et al. (2015), and Chun et al. (2018)	Prefrontal, Temporal, PHG, NAcc, Corticostriatal	Hyperactivation, abnormal connectivity	↓Cognitive control, emotion	Adolescents: PHG/ITG hyperactivity, abnormal connectivity	—
fNIRS	Zhang and Li (2025), Liu et al. (2023), and Li et al. (2023)	DLPFC, OFC, TPJ	Prefrontal inhibition, ↓creativity	↓Creativity, ↑impulsiveness	—	—
DTI	Hu et al. (2017)	SLF, Internal Capsule, Corona Radiata	↓FA, ↑MD	WM integrity deficit	—	—
DTI and MRI	Tymofiyeva et al. (2020)	Amygdala	Abnormal connectivity	—	Adolescents: ↑structural connectivity	Females: ↑SPD, ↑sleep/depression problems
fMRI and MRI	Schmitgen et al. (2022), Horvath et al. (2020), and Gao et al. (2025)	Prefrontal, Parietal, Insula, Temporal, Cerebellum, Thalamus	Abnormal connectivity, GMV/WMV alterations	↓Cognitive control, ↑impulsiveness/depression tendency	Adolescents: abnormal functional connectivity	—
	Tie et al. (2025) and Ko et al. (2023)	Prefrontal, Parietal, ACC, Angular gyrus, Insula, Amygdala, Hippocampus	Abnormal connectivity	Abnormal reward/emotion/memory	Adolescents: abnormal functional connectivity	—
MRI VBM	Montag et al. (2018), He et al. (2017), and Takeuchi et al. (2018)	Prefrontal, Temporal, NAcc, Insula	GMV/WMV alterations, functional connectivity	↓Cognitive control, abnormal reward/emotion/memory	Adolescents: more pronounced structural deviations	Females: ↑behavioral susceptibility
fMRI DTI VBM and MRI combined	Sadeghi et al. (2021)	Parietal, Temporal, Cerebellum, Insula, ACC	GMV/WMV alterations, hyperactivation	↓Cognitive control	—	Females: ↑Neuro-specific

This table systematically organizes key information for each study, including neuroimaging modalities, critical brain regions, primary findings, behavioral implications, and age/gender effects. Symbols (↑/↓) indicate the direction of change in brain activity or structure. ↑ Represents Increase, and ↓represents decrease.

WM, working memory; SN, salience network; DMN, default mode network; FPA, frontopolar area; TP, temporal pole; PHG, parahippocampal gyrus; ITG, inferior temporal gyrus; NAcc, nucleus accumbens; DLPFC, dorsolateral prefrontal cortex; OFC, orbitofrontal cortex; TPJ, temporoparietal junction; SLF, superior longitudinal fasciculus; FA, fractional anisotropy; MD, mean diffusivity; GMV, grey matter volume; WMV, white matter volume; ACC, anterior cingulate cortex; SNSA, social networking site.

Functionally, the data reveal a profile of context-dependent dysregulation. Fourteen studies provide convergent evidence that PFC activity is not merely reduced, but functionally compromised. Specifically, during tasks requiring creative cognition or risk evaluation, the DLPFC and OFC exhibit hypoactivation or maladaptive dysregulation (Li et al., 2023; Liu et al., 2023; Zhang and Li, 2025). Behaviorally, this manifests as a diminished capacity for inhibitory control and a heightened sensitivity to potential losses. At the network level, this focal dysfunction expands into a “disconnection syndrome,” characterized by widespread functional decoupling within the prefrontal-parietal control network and the DMN, as well as aberrant ACC recruitment under cognitive load (Darnai et al., 2019; Liu et al., 2025; Su et al., 2021; Wang M. et al., 2019). In adolescents specifically, the breakdown is even more fundamental: reduced functional connectivity in cortical-striatal circuits suggests a failure to integrate top-down executive commands with bottom-up motivational drives (Lin et al., 2015; Yao et al., 2025).

Structurally, the nature of PFC alteration is heavily modulated by biological maturity. In Adults, structural changes tend to be characterized by focal plasticity. While volumetric reductions are frequently observed in emotion-regulation hubs like the ACC and amygdala (He et al., 2017; Montag et al., 2018), there is also evidence of potential compensatory expansion in valuation regions such as the OFC and PHG (Gao et al., 2025; Sadeghi et al., 2021), reflecting experience-dependent remodeling.

In Adolescents, The impact appears more diffuse and disruptive. Takeuchi et al. (2018) provided compelling evidence that frequent internet usage correlates with impeded GMV development across extensive prefrontal regions involved in language, executive function, and emotional processing. This implies that excessive digital engagement may arrest the normative structural maturation (e.g., synaptic pruning) of these critical cortical areas during a sensitive developmental window.

Collectively, although specific patterns vary by task paradigm and addiction subtype, the compromise of the PFC emerges as a robust, cross-modal finding. These results establish prefrontal dysfunction as a transdiagnostic neural marker of IA. Theoretically, this substantiates the Dual-Process Model, representing the physiological tipping point where deficits in top-down control fail to regulate bottom-up striatal urges.

### 3.6 Gender-related differences in behavioral and neural findings

Behavioral and neuroimaging findings consistently underscore that gender acts as a critical moderator in the pathology of IA, demarcating distinct etiological pathways driven by divergent usage motivations and platform preferences. Behaviorally, the literature delineates a clear sexual dimorphism in susceptibility. Males typically exhibit heightened reward responsiveness and addiction tendencies triggered by gaming-related cues, reflecting a pursuit of achievement and sensation seeking. Conversely, females demonstrate a higher propensity for social media and SVA, behaviors largely driven by needs for emotional communication, social maintenance, and stress relief (He et al., 2017; Sadeghi et al., 2021; Tymofiyeva et al., 2020; Walla and Zheng, 2024). Clinically, this manifests as a higher burden of internalizing symptoms in females with IA, including social anxiety, depression, and sleep disturbances.

These behavioral divergences are anchored in distinct neural signatures. In females, brain abnormalities are predominantly concentrated in regions subserving emotion regulation and social cognition, suggesting a vulnerability in the affective network. Specifically, addiction severity in females correlates with increased GMV in the left anterior insula (Sadeghi et al., 2021) and volumetric deficits in the bilateral amygdala (He et al., 2017), alterations that likely provide the biological substrate for the emotional dysregulation fueling social networking addiction. In contrast, males exhibit more pronounced disruptions in WMI and functional networks associated with impulse control and executive execution (Tymofiyeva et al., 2020). This structural compromise aligns with the high-impulsivity profile often observed in male-predominant gaming disorders, pointing to a deficit in the “executive-control” circuitry.

### 3.7 Summary of findings

In summary, this review elucidates the complex neurobiological architecture of IA, revealing a landscape defined by both shared mechanisms and distinct moderators. The PFC emerges as the transdiagnostic core of dysfunction. Across all addiction subtypes, consistent functional and structural deficits in the DLPFC, OFC, and ACC point to a universal failure in the top-down regulation of reward and inhibition. However, this core dysfunction is significantly modulated by biological maturity. Adolescents display widespread, diffuse network disruptions, particularly in cortico-striatal circuits, which interfere with ongoing neurodevelopment and structural maturation, whereas adults exhibit more localized, experience-dependent remodeling associated with habit formation. Furthermore, neural correlates are stratified by gender, with females exhibiting alterations in emotion-processing networks (such as the insula and amygdala) and males showing deficits in impulse-control circuits. This distinction likely reflects the specific cognitive and affective demands of their preferred digital platforms, such as the need for emotional engagement in social media versus competitive impulsivity in gaming. Collectively, these findings challenge a “one-size-fits-all” model, underscoring the urgent need for precision psychiatry approaches that tailor interventions to the specific developmental stage and gender-specific neurocognitive profiles of the individual.

## 4 Discussion

This scoping review synthesizes evidence from 28 empirical studies to elucidate the neurobiological footprint of IA across diverse behavioral phenotypes, including SVA, SPA, and SNSA. Moving beyond a fragmented view of isolated brain deficits, our synthesis reveals that IA represents a system-wide reorganization of large-scale brain networks. Specifically, the pathology is best characterized by a breakdown in the dynamic interaction among the “Triple Networks”: the DMN, the ECN, and the SN. Furthermore, we posit that these neural alterations are not uniform; rather, they are distinctively modulated by developmental stage and gender, where the former represents a conflict between normal maturation and pathological disruption and the latter reflects distinct affective versus impulsive pathways.

### 4.1 Functional brain alterations: the “triple network” dysregulation

The core functional pathology of IA can be understood as a failure in how major brain networks coordinate with one another. This dysregulation manifests primarily as a specific triad of deficits: maladaptive self-focus within the DMN, compromised top-down regulation in the ECN, and sensitized reward evaluation in the Salience systems.

#### 4.1.1 DMN hyperactivation and the introspective Bias

A consistent neurophysiological signature of IA is the persistent over-activation of the DMN (including the MPFC, PCC, and dMPFC) when individuals are exposed to addiction-related cues. Theoretically, this hyperactivity underpins an introspective bias in which attention is maladaptively directed inward to process immediate gratification, specifically social validation or digital feedback, rather than focusing on external real-world tasks (Bellana et al., 2017; Xu et al., 2016). This creates a state of resource competition, where the brain’s limited energy is monopolized by self-referential thinking, effectively suppressing the cognitive capacity required for goal-directed behavior (Fox et al., 2015; Leech et al., 2011). Critically, stress appears to act as a catalyst in this circuit. Continuous rumination amplifies the perception of external stressors, which depletes the cognitive reserves needed for self-control and further entrenches digital escapism as a primary, albeit maladaptive, coping mechanism (Song and Park, 2019).

#### 4.1.2 Impaired inhibitory control and prefrontal dysregulation

The failure of self-regulation in IA is underpinned by a functional dissociation within the ECN. During standard inhibitory control tasks, individuals with IA consistently exhibit hypoactivation in the DLPFC and ACC, often accompanied by compensatory theta band activity (Dong et al., 2012). This pattern indicates a fundamental deficit in conflict monitoring and the suppression of prepotent urges.

However, this deficit is context-dependent. Recent evidence reveals that under conditions of risk or loss, the DLPFC and sensorimotor regions exhibit significant hyperactivation (Liu et al., 2023). This unmasks a maladaptive dissociative pattern: a weakened capacity for active impulse suppression coexists with a heightened, stress-induced reactivity to negative feedback. Notably, Liu et al. (2023) specifically identified this abnormal cerebellar and sensorimotor recruitment within the context of SVA, attributing it to an “over-alert” state driven by rapid feedback loops. However, we interpret this finding as indicative of a broader mechanism wherein loss signals precipitate avoidance behaviors rather than rational adjustment. This drives the individual to persist in usage to escape the distress of potential losses. While this mirrors deficits seen in substance addiction (Ekhtiari et al., 2017), withdrawal in IA is distinctively triggered by environmental cues (e.g., notifications) rather than purely physiological dependence, highlighting a cognitive-emotional reinforcement loop.

#### 4.1.3 Reward, emotion, and feedback processing dysregulation

The maintenance of addictive behavior is further driven by the sensitization of the mesolimbic reward system and impaired outcome evaluation. Upon exposure to digital cues, individuals with IA demonstrate hypersensitivity in dopaminergic regions, including the VTA and NAcc, correlating with elevated physiological arousal (Balconi et al., 2017a). This reward system hyperactivity is inextricably linked to emotional dysregulation, particularly in adolescents where negative emotional states, such as those resulting from bullying or lack of family support, drive emotional compensation seeking through virtual platforms (Boniel-Nissim and Sasson, 2018; Hossain et al., 2024). This behavioral pathway is reinforced by neuroendocrine alterations; elevated cortisol levels in IA correlate negatively with prefrontal-striatal connectivity, suggesting that chronic stress may “hijack” reward circuits to prioritize immediate relief over top-down control (Dedovic et al., 2009). Temporally, this dysregulation is evidenced by blunted FRN amplitudes and abnormal P300 potentials (Balconi et al., 2017a; Kallen et al., 2020). These electrophysiological markers indicate a specific deficit in processing negative outcomes, creating a vicious cycle where individuals fail to adjust their behavior despite experiencing adverse real-world consequences.

### 4.2 Structural brain alterations: from plasticity to persistent vulnerability

#### 4.2.1 Gray matter volume changes

Structural MRI findings reveal that IA induces morphometric alterations across three synergistic systems, reflecting a combination of use-dependent plasticity and maladaptive atrophy. Within the reward and decision-making circuitry, increased GMV is frequently observed in the OFC and bilateral cerebellum (Gao et al., 2025). These volumetric increases likely represent experience-dependent plasticity resulting from the chronic engagement of risk/reward calculation and sensorimotor coordination inherent in digital usage. Conversely, regions critical for emotion regulation and self-control, such as the sgACC and amygdala, exhibit volume reductions (Montag et al., 2018). This atrophy correlates positively with addiction severity and impulsivity, particularly in social networking addiction, suggesting that structural deficits in limbic regulators underlie the heightened emotional lability in these individuals. Furthermore, in higher-order cognitive systems, reduced volume in the PHG, insula, and temporal lobes points to an impairment in memory and interoceptive integration. Longitudinal evidence is particularly concerning for adolescents, where high-frequency internet use is associated with widespread developmental delays in these cortical regions, suggesting that excessive digital engagement may physically impede the normative maturation of brain areas required for complex cognitive functions (Martínez-Iniesta et al., 2025).

#### 4.2.2 White matter integrity and developmental implications

Beyond gray matter, the microstructural integrity of white matter tracts serves as a critical indicator of neural efficiency. Although limited in number, DTI studies indicate that IA is associated with reduced FA and increased MD in tracts responsible for motor control and inter-hemispheric communication, such as the internal capsule and corona radiata (Hu et al., 2017; Wang et al., 2016). These structural disruptions imply a compromise in the efficiency of signal transmission between executive and sensory regions. For adolescents, who are in a critical window for axonal myelination, such damage is not merely static but developmental; it threatens to derail the trajectory of white matter maturation, potentially leading to persistent deficits in the higher-order cognitive coordination required for adult functioning (Takeuchi et al., 2018). Thus, the structural impact of IA extends from local gray matter remodeling to the disruption of global connectivity, establishing a biological basis for the persistent cognitive and behavioral vulnerabilities observed in this population.

### 4.3 The prefrontal cortex as a transdiagnostic neural hub

Although IA manifests through heterogeneous behaviors ranging from the sensory overload of short-video viewing to the social validation seeking of networking sites, the PFC consistently emerges as a convergent neural target. Systematic evidence across both adolescent and adult populations identifies widespread dysfunction within the PFC, specifically involving the DLPFC, OFC, and ACC. This ubiquitous involvement suggests that prefrontal dysregulation serves as a transdiagnostic neural signature of IA, representing the physiological intersection where impaired top-down cognitive control fails to regulate bottom-up reward urges. Structurally, the PFC exhibits a pattern of maladaptive remodeling; while adaptive volume increases in the OFC may reflect enhanced reward valuation processing, the concurrent atrophy in regulatory regions like the sgACC compromises the “braking system” of brain. Functionally, this manifests as a critical failure in network integration: the PFC is unable to effectively modulate the hyperactive DMN and reward circuits, leading to the prioritization of immediate digital gratification over long-term goals. Consequently, the PFC represents the most viable candidate for targeted interventions. Therapeutic strategies, including cognitive bias modification and non-invasive brain stimulation (e.g., TMS), should specifically aim to restore prefrontal plasticity, thereby re-establishing the hierarchical control necessary to inhibit compulsive usage.

### 4.4 Developmental differences: neural mismatch vs. adaptive remodeling

The neural mechanisms underpinning IA are not static but are significantly modulated by the developmental stage of the individual, reflecting a divergence between developmental disruption in adolescents and habitual remodeling in adults. In adolescents, IA pathophysiology is best framed within the Dual Systems Model of a developmental mismatch. The adolescent brain is characterized by the premature maturation of striatal reward systems relative to the prolonged development of prefrontal control networks. Our synthesis reveals that IA exacerbates this inherent biological vulnerability, leading to a hyper-coupling of emotion-movement-reward circuits and reduced cortico-striatal connectivity (Ye et al., 2020; Dedovic et al., 2009). Because this occurs during a critical window of neuroplasticity, excessive internet exposure does not merely alter function but threatens to derail the normative ontogenetic trajectory of white matter myelination and gray matter refinement, potentially embedding long-term deficits in executive function. In contrast, the adult brain possesses established control architectures. Neural alterations in adults, manifesting primarily as reduced efficiency in the ECN and rigid DMN engagement, likely represent adaptive remodeling following prolonged, chronic exposure rather than developmental arrest. This distinction implies that while adult IA may be driven by entrenched habit formation requiring unlearning strategies, adolescent IA represents a deviation in neurodevelopment requiring protective interventions to realign maturation trajectories.

### 4.5 Gender differences: distinct motivations and neural signatures

Gender acts as a critical moderator in the pathology of IA, shaping not only behavioral phenotypes but also the underlying neural recruitment patterns. Behavioral evidence consistently indicates a divergence in usage motivation: females demonstrate stronger susceptibility to social media and SVA driven by needs for emotional connection and social validation, whereas males are more prone to gaming addiction driven by achievement and competitive motives. These distinct behavioral pathways are mirrored by gender-specific neural signatures. Females exhibit greater plasticity and structural anomalies in regions central to socio-emotional processing, such as the left anterior insula and amygdala (Sadeghi et al., 2021). This specific neural vulnerability aligns with the higher comorbidity of internalizing disorders, such as depression and anxiety, observed in female IA populations. Conversely, males exhibit more pronounced structural abnormalities in white matter tracts (e.g., corpus callosum) and sensorimotor networks, reflecting the high-intensity visuomotor demands and impulse-control deficits associated with gaming. We propose a “Bio-Social Feedback Model” to explain this dichotomy, that biological predispositions (e.g., higher social sensitivity in females vs. impulsivity in males) synergize with sociocultural cues and specific digital content preferences to reinforce distinct neural circuits. Consequently, the “brain of IA” is not uniform across genders; rather, neural alterations reflect the specific cognitive and emotional demands of the preferred digital platform. This underscores the necessity for precision medicine approaches, where interventions for females might prioritize emotional regulation and social cognition, while those for males focus on impulse control and behavioral inhibition.

## 5 Practical implications and future research

IA prevention requires multi-level strategies. Individually, proactive behavioral regulation and offline engagement are vital. At family and school levels, early identification of high-risk youth and digital literacy training are essential. Critically, interventions should be gender-tailored: emphasizing emotional regulation and social support for females (social media-related), while focusing on cognitive-behavioral therapy and executive control for males (gaming-related). Societally, strengthened platform governance and age-appropriate regulations are needed.

Future research should prioritize: (1) longitudinal designs to clarify causal relationships and neural plasticity during critical developmental windows; (2) multimodal integration and cross-subtype comparisons (for instance, gaming vs. social media) to identify objective biomarkers; and (3) investigation of the socio-biological drivers of gender differences. Standardizing diagnostic criteria and neuroimaging parameters remains essential to enhance comparability across studies.

In summary, this scoping review integrates behavioral and neuroimaging evidence within a developmental and gender-sensitive framework, providing a roadmap for precision-based prevention and intervention strategies.

## 6 Conclusion

This scoping review consolidates a decade of neuroimaging evidence, delineating the neurobiological footprint of IA not as a focal lesion but as a system-wide reconfiguration of brain organization. Our synthesis highlights three convergent findings. First, the pathology of IA is fundamentally characterized by a breakdown in the dynamic interaction among the “Triple Networks,” with the PFC emerging as the transdiagnostic epicenter of dysfunction. Second, these neural alterations manifest multimodally, involving widespread structural remodeling and functional connectivity disruptions that extend from cortical hubs to subcortical and cerebellar regions. Third, and perhaps most critically, we establish that the “brain of IA” is not monolithic; rather, its neural expression is distinctively modulated by developmental stage (reflecting a conflict between maturation and derailment) and gender (reflecting distinct affective versus impulsive pathways).

Theoretically, this work advances the field by integrating fragmented regional findings into a coherent the Control-Reward-Emotion framework. By explicitly disentangling the moderating effects of biological sex and developmental timing, this review addresses the limitations of prior age-mixed analyses and provides a foundational model for gender-sensitive neurocognitive profiles.

However, several limitations warrant a cautious interpretation of these findings. The current literature is constrained by sample representativeness, with a predominant focus on adolescents and young adults (ages 10–30), potentially limiting generalizability to older populations. Furthermore, the reliance on cross-sectional designs hinders the ability to distinguish whether neural anomalies are the cause or the consequence of addiction. Methodological heterogeneity across imaging modalities and diagnostic criteria also poses challenges for quantitative meta-analysis. Consequently, future research must prioritize longitudinal designs and standardized protocols. Ultimately, elucidating these specific neural trajectories is the critical next step toward moving beyond generic treatments and establishing a framework for Precision Psychiatry, tailoring intervention strategies to the specific neurodevelopmental and gender-specific profile of the individual.

## Data Availability

The original contributions presented in the study are included in the article/Supplementary material, further inquiries can be directed to the corresponding authors.
